# Supplementary material for: Explaining machine-learning models for gamma-ray detection and identification
Source: PLoS One. 2023 Jun 20;18(6):e0286829. doi: 10.1371/journal.pone.0286829 (PMC10281578; doi:10.1371/journal.pone.0286829)
Supplement: Attachment — Submitted filename: response_v6.pdf [file pone.0286829.s001.pdf]

Responses to the editor and reviewers

We thank all three reviewers and the editor for their thoughtful and helpful comments. We have done our best to address them and summarize these changes here. As requested, we are providing both the updated manuscript as well as a version of the manuscript highlighting the differences.

Editor's Comments

Editor Point P 1 — 1. Please ensure that your manuscript meets PLOS ONE's style requirements, including those for file naming. The PLOS ONE style templates can be found at https://journals.plos.org/plosone/s/file?id=wjVg/PLOSOne_formatting_sample_main_body.pdf and https://journals.plos.org/plosone/s/file?id=ba62/PLOSOne_formatting_sample_title_authors_affiliations.pdf

Reply: We updated the manuscript in the following ways:

- The affiliation addresses were put in the correct format.
- Figure references in the text now use "Fig".
- Figure filenames were changed from `fig#.eps` to `Fig#.eps`
- Numbers were removed from all of the headings in the "Explanation methods" section.
- References in the text to other sections was changed from references by number to references by name, although we could not find specific guidance for the correct or preferred way of cross-referencing within the text.

Editor Point P 2 — 2.We note that the grant information you provided in the 'Funding Information' and 'Financial Disclosure' sections do not match.

When you resubmit, please ensure that you provide the correct grant numbers for the awards you received for your study in the 'Funding Information' section.

Reply: Thank you, we realized that the "grant number" we listed in the Funding Information section is only an internal code and so we have removed it. The relevant funding details (as DOE labs funded by the DOE) are our DOE contract numbers. The funding statement required by our organizations and the DOE, which is clarified in the next question and updated in our new cover letter, is now also correct.

Editor Point P 3 — 3. Thank you for stating the following in the Acknowledgments Section of your manuscript:

"The project was funded by the U.S. Department of Energy, National Nuclear Security Administration, Office of Defense Nuclear Nonproliferation Research and Development."

We note that you have provided funding information that is not currently declared in your Funding Statement. However, funding information should not appear in the Acknowledgments section or other areas of your manuscript. We will only publish funding information present in the Funding Statement section of the online submission form. Please remove any funding-related

text from the manuscript and let us know how you would like to update your Funding Statement. Currently, your Funding Statement reads as follows:

“This work was performed under the auspices of the U.S. Department of Energy by Lawrence Berkeley National Laboratory (LBNL) under Contract DE-AC02-05CH11231. The project was funded by the U.S. Department of Energy, National Nuclear Security Administration, Office of Defense Nuclear Nonproliferation Research and Development.

This manuscript has been authored in part by UT-Battelle, LLC, under contract DE-AC05-00OR22725 with the US Department of Energy (DOE). The US government retains and the publisher, by accepting the article for publication, acknowledges that the US government retains a nonexclusive, paid-up, irrevocable, worldwide license to publish or reproduce the published form of this manuscript, or allow others to do so, for US government purposes. DOE will provide public access to these results of federally sponsored research in accordance with the DOE Public Access Plan (http://energy.gov/downloads/doe-public-access-plan).

The funders had no role in study design, data collection and analysis, decision to publish, or preparation of the manuscript.”

Please include your amended statements within your cover letter; we will change the online submission form on your behalf.

Reply: We have removed funding information from the Acknowledgments, and have added our current statement to the cover letter.

Editor Point P 4 — 4. We note that you have stated that you will provide repository information for your data at acceptance. Should your manuscript be accepted for publication, we will hold it until you provide the relevant accession numbers or DOIs necessary to access your data. If you wish to make changes to your Data Availability statement, please describe these changes in your cover letter and we will update your Data Availability statement to reflect the information you provide.

Reply: We do not wish to change the Data Availability statement. We are ready to provide access to the code and data, the repository is now live at https://gitlab.com/lbl-anp/radai/explanations, and the data has been submitted to Dryad, with DOI https://doi.org/10.7941/D1XC97. While the paper is in peer review, the data will not be downloadable from the DOI link, but reviewers will be able to download it at this link: https://datadryad.org/stash/share/LejhwLscutrY9Q1jeBvmrCRMRPP6PZFrCy4DezgMLLE.

Editor Point P 5 — Additional Editor Comments: Kindly make sure that appropriate machine learning evaluation methods are used and reported. The paper will not be accepted without the proper evaluation of the proposed work.

Reply: Thank you, we have considered all of the reviewers’ comments but saw some of their suggestions as beyond the scope of the manuscript and peripheral to its focus, which is to ensure that these explanation methods are clearly articulated in this problem space. In principle, explanation methods work even for a bad model; the explanations will just not be very helpful. So it is convenient for demonstration purposes that our model be adequate or even good, but the model itself can be a “black box” for our preferred methods of LIME and SHAP.

Additionally, within the specific problem area we are working (gamma-ray spectral detection and identification), there are not yet clearly established performance baselines that are agreed upon by the community. For example, it is known due to the Poisson nature of the data that we cannot expect 100% accuracy, but not what level of accuracy we should expect a model to achieve for, e.g., a set of Cs-137

sources encountered in a complex urban environment. We are engaged in this work elsewhere, but for our purposes here it means that we have reason to believe our models perform quite well, but we cannot easily say how well it performs compared to a theoretical maximum. (This situation can be distinguished from spectral identification scenarios with more stable background and high signal-to-noise, where 100% accuracy can be expected, such as in references 11 and 18, among others.)

Reviewer 1

Reviewer Point P 1.1 — Major-1) A workflow of the procedure is needed to better understand the work done. A graphical schematization of the ANN used would also be appreciable.

Reply: In the "Data and model" section we explain how the data were prepared ("The dataset and its preparation") and how individual models were trained and over what hyperparameter space ("Model for detection and identification"). We would be interested in knowing what additional details the reviewer thinks are necessary.

We did not provide a schematic of the final network because we were hesitant to go into too much detail about the model in the main body of the paper lest it distract from the point, which is to explain the explanation methods. We included a table of model parameters because one could take these and input them into Keras nearly verbatim and recreate our model, whereas in our experience the finer model details tend to be lost in diagrams. Additionally, the model itself is now available in the code repository, and anyone can download and examine it.

Reviewer Point P 1.2 — Major-2) It's not very clear to me how you set the network parameters. In my opinion a cross validation procedure is needed to rule out possible neural network overfitting issues.

Reply: The reviewer is correct that cross validation would further improve the model search and guard against overfitting, but we don't believe that level of rigor is necessary for our purposes, since we were not focused on having the best model, but just a good model.

We did notice while we were thinking about this point that we wrote that we did a "brute-force" search, but technically it was a random search since we did not visit every single hyperparameter combination, and we have updated the text to reflect that.

Reviewer Point P 1.3 — Major-3) Once the cross validation procedure is implemented it would be better to put the average performance with uncertainties inside the confusion matrix.

Reply: We have updated the confusion matrix to show percentages for greater clarity as suggested by reviewer 3, but we view implementing the procedures suggested and showing uncertainties as beyond the scope of this work.

Reviewer Point P 1.4 — Major-4) The confusion matrix is fine since you have many classes but it would be good to provide some overall performance through AUC and accuracy as well.

Reply: We had left out mention of the overall accuracy of the model, so we added that information. We would much prefer to leave more detailed model performance information to a later publication focused on model development, not one focused on explanations.

Reviewer Point P 1.5 — Minor-1) What version of keras and tansor flow did you use? Through which framework?

Reply: We used Tensorflow and Keras version 2.11.0 and have added that information to the paper. We wrote a custom Python script to generate and train our models.

Reviewer 2

Reviewer Point P 2.1 — The authors say that the code and datasets will be available upon publication at publication, which is great! But it's not there yet: gitlab returns a 404: so the "no" to question #3 above is a qualified one: at this moment it's a "no", but it would be a "yes" if the authors follow through. I would have loved to poke around in it as part of this review.

Reply: Thank you, we were not prepared to release the code and data at the time of submission but have since done so. Please visit the repository at https://gitlab.com/lbl-anp/radai/explanations and try out the explanations with our data, which are downloadable for the reviewers at https://datadryad.org/stash/share/LejhwLscu [illegible]
We have provided Jupyter notebooks demonstrating the explanations we have evaluated in this manuscript, as well as the Tensorflow model demonstrated in the paper and alternate models, and we welcome any feedback on those tools and their presentation there.

Reviewer 3

Reviewer Point P 3.1 — 1- The architecture used for modeling the data is inspired by references, 12, 18 and 19. Have authors tried other architectures or this is the only one? Given the model agnostic nature of most explanation techniques, would be informative if the authors reported their observation, on two different architectures. Given the availability and effectiveness of AutoML models, this can be done in a short time.

Reply: Thank you, this is a good point. We could not think of a way to easily include the results of multiple models without making the manuscript longer and more complex, but we thought that training some other models (e.g., multiple fully-connected layers similar to those used in reference 11, different pre-processing methods such as logarithmic scaling) and providing them with the released code would be a reasonable solution, so the reader could try using the explanation tools those other models, or even with their own models trained with the data provided.

In addition, while doing this, we noticed that for logarithmic scaling of the data, the results from saliency mapping may be improved. A note to this effect was added to the Results section. This result underscores how these methods cannot necessarily be used "out of the box" without thorough testing.

Reviewer Point P 3.2 — 2- Authors discuss cases from higher a and lower ends of the energy spectrum along with a more complex case with overlapping signature. I personally think, including an example of misclassification by the model, also has value to demonstrate how the model comes up with the wrong decision.

Reply: We thought this was an excellent suggestion, and so we added a second example where Xe-133 is incorrectly identified as Tl-201, which involved adding the new Fig. 8 as well as discussion in the text.

Along with this change, we added percent confidence numbers on Fig. 7 (and the numbers appeared to have changed slightly, they were likely from the model we used when drafting the paper, which had very similar performance to the final model). In addition, we made a minor change in wording to the caption of Fig. 7.

We also thought it would be appropriate to add a citation to the National Nuclear Data Center for the various gamma-ray decay data used in the text.

Reviewer Point P 3.3 — 3- A normalized confusion matrix in lieu of, or accompanying figure 3 would make it easier to understand model performance.

Reply: We have replaced Fig. 3 with a normalized confusion matrix. Please note when understanding performance that 100% accuracy is not expected for each category since this domain includes both detection (i.e., determining whether a source is present versus background) and not just identification of a source.

Other edits

In addition to the changes described above, we made the following changes:

- Minor wording changes were made to the Abstract and Introduction.
- An Acknowledgment was added to thank Brian Quiter for suggestions.
- The status of reference [44] was corrected to "in preparation."
- While preparing the code and notebooks for the repository, we noticed that whenever we calculated counterfactuals we have always calculated the explanations $\boldsymbol{\phi}_1$ and $\boldsymbol{\phi}_2$ using the same random seed, but that had not been mentioned. In fact, we found that the counterfactuals can be much less clear if one uses different random seeds. This effect presumably results from making a relatively small number (thousands) of random samples of a high-dimensional space (2^M, and we used $M = 64$) and fitting a low-dimensional representation, so there is some noise to the fit. Using different random samples for two such fits adds extra noise that muddles the comparison. We have added a note to the text mentioning this point.
